# Molecular Targeted Therapy in the Management of Recurrent and Metastatic Nasopharyngeal Carcinoma: A Comprehensive Literature Review

**DOI:** 10.7759/cureus.4210

**Published:** 2019-03-09

**Authors:** Abdulaziz A Almobarak, Alaeddin B Jebreel, Ahmed Abu-Zaid

**Affiliations:** 1 Otolaryngology, Alfaisal University College of Medicine, Riyadh, SAU; 2 Otolaryngology, King Faisal Specialist Hospital and Research Centre, Riyadh, SAU; 3 Oncology, Alfaisal University College of Medicine, Riyadh, SAU

**Keywords:** nasopharyngeal carcinoma, recurrent, metastatic, molecular targeted therapy, vegf, egfr

## Abstract

Nasopharyngeal carcinoma (NPC) is the most frequent malignancy arising in the nasopharynx. NPC, to a larger degree, substantially differs from the other malignancies of the head and neck, in terms of incidence, etiology, risk factors, molecular pathogenesis, clinical behavior, management and prognosis. Fundamentally, the management of NPC is entirely guided by the disease stage. Favorably, patients with early- stage disease have encouraging survival outcomes with stand-alone radiation therapy (RT), specifically following the emergence of intensity-modulated RT (IMRT). The reported five-year local control rates are outstanding, and they range from 70% to 90%. Unfortunately, around one-third (30%) of patients presents with loco-regional or distant recurrences, despite rigorous curative treatment in the intermediate (stage II) and advanced (stage III-IVB) NPC disease. At the present time, the management of recurrent and metastatic NPC is largely discouraging and presents significant challenges to the treating physicians. Broadly speaking, there are three management schemes utilized in the management of recurrent and metastatic NPC, namely: (i) palliative systemic chemotherapy, (ii) molecular targeted therapy, and (iii) immunotherapy. The goal of this study is to holistically review the existing body of literature on the utility and safety of molecular targeted therapy in the management of patients with recurrent and metastatic NPC, with a special focus on vascular endothelial growth factor (VEGF) and epidermal growth factor receptor (EGFR) targets.

## Introduction and background

Nasopharyngeal carcinoma (NPC) is a malignancy that originates from the epithelial lining of the nasopharynx. The etiology of NPC is largely multifactorial, involving genetic susceptibility, Epstein-Barr virus (EBV) infection and an array of environmental risk factors (for example, smoking and high consumption of preserved food) [1]. Histologically, NPC is categorized into three types, namely: (i) keratinizing squamous cell carcinoma (World Health Organization [WHO] type I), (ii) differentiated (WHO II) or undifferentiated (WHO type III) non-keratinizing squamous cell carcinoma, and (iii) basaloid squamous cell carcinoma [2]. In endemic regions (such as China, Korea and Taiwan), the undifferentiated non-keratinizing squamous cell carcinoma (WHO type III) is the most predominate histological type, and it is strongly linked to EBV infection. Conversely, in non-endemic regions, keratinizing squamous cell carcinoma is the most frequent histological type (WHO type I) [2-3].

NPC is clinically staged in accordance with the Tumor, Node, Metastasis (TNM) system (8th edition) that is jointly developed by the American Joint Committee on Cancer (AJCC) and the Union for International Cancer Control (UICC) [4]. Therapeutic management of NPC is fundamentally based on the disease stage [5]. The universal agreed upon consensus is to manage early-stage disease (stage I) with radiotherapy (RT) alone; intermediate stage (stage II) with RT with/without concurrent chemotherapy (CRT); and advanced stage (stage III-IVB) with upfront CRT [4-6].

Patients with early-stage disease have favorable clinical and survival outcomes with stand-alone RT, particularly after the introduction of intensity modulated radiotherapy (IMRT). It has been estimated that the five-year local control rates of T3 and T4 diseases are 90% and 75-80%, respectively [6]. However, around 5-15% and 15-30% of patients will develop loco-regional or distant treatment failures, respectively [6]. Furthermore, nearly 50% of patients with an obvious local recurrence have concurrent distant metastatic foci elsewhere [7-8]. In addition, around 30% of patients with stage III-IVB disease will experience distant recurrence following administration of intensive concurrent CRT [2, 6].

Treatment of recurrent and metastatic NPC is disappointing and challenging, too [2, 6]. Generally, there are three employed treatment modalities in the management of recurrent and metastatic NPC, namely: (i) palliative systemic chemotherapy, (ii) molecular targeted therapy, and (iii) immunotherapy [2].

The primary objective of this study is to provide a comprehensive up-to-date literature review on the role of molecular targeted therapy in the management of patients with recurrent and metastatic NPC, with a special focus on vascular endothelial growth factor (VEGF) and epidermal growth factor receptor (EGFR) targets.

## Review

Literature search

The PubMed® database engine was reviewed until 31st December 2018 using the following keywords: “recurrent”, “metastatic”, “nasopharyngeal carcinoma” and “targeted therapy”. Only English-published studies were included. Further references from published articles were also manually screened for potential additional studies. The study inclusion criteria included: (i) patients with recurrent or metastatic NPC, (ii) studies reporting clinical retrospective cohorts or trials, and (iii) studies reporting molecular targeted therapies against VEGF and EGFR. The study exclusion criteria included: (i) patients with primary locally advanced NPC, (ii) pre-clinical studies, and (iii) studies not reporting molecular targeted therapies against VEGF and EGFR.

For each included study, the following details, whenever available, were retrospectively reviewed including authors, year of publication, study type, study sample size, type of molecular targeted therapy, clinical efficacy, toxicity profile and survival outcomes.

Inhibition of vascular endothelial growth factor

Sorafenib, pazopanib, famitinib, sunitinib and axitinib are multi-targeted tyrosine kinase inhibitors (TKIs) of VEGF receptor (VEGFR). The VEGF-VEGFR interaction activates a signaling cascade that promotes angiogenesis, tumor growth and metastasis [9-10]. It has been shown that NPC is characterized by high expression of VEGF, which in turn is adversely correlated with poor survival [11]. Additionally, NPC is characterized by high expression of endostatin, an endogenous angiogenesis inhibitor, in around 20% of the NPC cases [12]. The proposed anti-angiogenic mechanism of action of endostatin is to block the pro-angiogenic actions of growth factors, particularly VEGF [13]. Endostar is a recombinant endostatin drug that functions as an anti-VEGF antibody [12]. Thus, the development of therapies geared toward molecular targeting of VEGFR and boosting the circulating levels of endostatin are plausible anti-angiogenic therapies in the management of patients with recurrent and metastatic NPC.

In 2007, Elser et al. (phase II trial) examined the efficacy and safety of single-agent sorafenib in seven patients with recurrent or metastatic NPC [14]. The median time to progression (TTP) and overall survival (OS) were 3.2 months and 7.7 months, respectively. The drug was well-endured with few grade III adverse events. No grade IV toxicities were reported. One patient died as a consequence of nasopharyngeal hemorrhage that was likely attributable to the underlying malignancy; the death was not related to the sorafenib treatment. The study concluded that sorafenib monotherapy was associated with modest clinical efficacy and acceptable drug-related safety profile in patients with recurrent or metastatic NPC.

In 2011, Lim et al. (phase II trial) investigated the clinical efficacy of monotherapy pazopanib in 33 Asian patients with recurrent or metastatic NPC who previously failed at least one line of chemotherapy [15]. Four (12.1%) patients were not evaluable for clinical efficacy, and 29 (87.9%) patients were evaluable for clinical efficacy. Partial response, stable disease and progressive disease were achieved in two (6.1%), 16 (48.5%) and 11 (33.3%) patients, respectively. The median TTP and OS were 4.4 months and 10.8 months, respectively. The one-year progression-free survival (PFS) and OS rates were 13% and 44.4%, respectively. The most frequent grade III-IV drug-related side effects included fatigue (15.2%), hand-foot syndrome (15.2%), anorexia (9.1%) and diarrhea/vomiting (6.1%). Two death events occurred due to myocardial infarction and uncontrolled epistaxis. Serial dynamic-contrast enhanced computed tomography (DCE-CT) studies exhibited a substantial decrease in tumor blood flow. The study concluded that pazopanib appeared to exhibit therapeutic benefits and tolerability in previously chemotherapy-pretreated patients with recurrent or metastatic NPC.

In 2011, Hui et al. (phase II trial) assessed the efficacy and safety of monotherapy sunitinib in 14 patients with recurrent or metastatic NPC. All patients previously failed platinum-based chemotherapy and received curative high-dose RT in the nasopharynx region [16]. Clinical benefits occurred in four (28.6%) patients, as follows: one (7.1%) patient achieved partial response for an interval of 5.6 months, and three (21.4%) patients achieved stable disease lasting for a minimum of 12 weeks. The median follow-up was 23.1 months. The median TTP, PFS and OS were 4.4 months, 3.5 months and 10.5 months, respectively. The one-year OS rate was 35.7%. Drug-related hemorrhagic adverse events occurred in nine (64%) patients, as follows: two patients with hematemesis, three patients with hemoptysis and six patients with epistaxis. Previous RT exposure to the chest was statistically significantly correlated with hemoptysis (p<0.05). Within the first cycle of sunitinib administration, two death-related events occurred. Both patients had local tumor invasion into the carotid sheath and developed lethal epistaxis/hematemesis, most likely attributable to the internal carotid rupture following tumor retraction. The study concluded that sunitinib was associated with modest clinical benefits in heavily chemotherapy-pretreated patients with recurrent or metastatic NPC. However, it was also concluded that sunitinib was adversely associated with an increased frequency of upper aerodigestive tract hemorrhagic events in NPC patients who received previous high-dose RT in the nasopharynx/neck region. It was speculated that the existing direct blood vessel invasion by the tumor mass was the major contributing risk factor in the development of severe bleeding events [17].

In 2013, Huang et al. (phase II trial) evaluated the clinical efficacy and toxicity of monotherapy famitinib in 58 Chinese patients with recurrent or metastatic NPC who previously failed at least two lines of chemotherapy [18]. Partial response and stable disease maintained for more than 12 weeks occurred in five (8.6%) and 16 (27.6%) patients, respectively. The median PFS was 3.2 months. Mild to moderate grade I-II side effects with supportive treatment were observed. The most commonly documented blood-related side effects comprised leucopenia, neutropenia and thrombocytopenia. On the other hand, non-blood-related side effects comprised hand-foot syndrome, proteinuria and hypertension. The frequency of severe grade III-IV side effects was minimal. The study concluded that famitinib exhibited therapeutic advantages and endurable drug profile in heavily chemotherapy-pretreated patients with recurrent or metastatic NPC.

In 2013, Xue et al. (phase II trial) explored the efficacy and safety of sorafenib combined with cisplatin and 5-fluorouracil (5-FU) in 54 patients with recurrent or metastatic NPC [19]. After a maximum of six schedules of combination chemotherapy, patients received maintenance monotherapy with sorafenib. A total of 42 (77.8%) patients achieved objective response rate (ORR), as follows: 41 patients with partial responses and one (n=1) patient with complete response. The median PFS and OS were 7.2 months and 11.8 months, respectively. Several drug-related side effects were observed, and they were consistent with the known toxicities associated with sorafenib, cisplatin and 5-FU. The top five most frequently documented adverse events (mild-to-moderate) included hand-foot-skin reactions (83.3%), leucopenia (77.8%), anemia (74.1%), anorexia (74.1%) and nausea (64.8%). Hemorrhage-related adverse events occurred in 12 (22.2%) patients, with one related death event. Additional drug-related serious adverse events included severe hyponatremia (n=1) and severe vomiting (n=1). The study concluded that the combination therapy of sorafenib, cisplatin and 5-FU was clinically feasible, effective and fairly tolerable in patients with recurrent or metastatic NPC. 

In 2013, Jin et al. (phase II trial) scrutinized the efficacy and safety of endostatin combined with gemcitabine and cisplatin (GC) chemotherapy in 30 patients with metastatic NPC [20]. The interval of follow-up ranged from 2.9 to 20.7 months (median 13.1 months). Only 28 patients were evaluable for response. Objective clinical response occurred in 24 (85.7%) patients including 14 and 10 patients with complete and partial responses, respectively. The median PFS, one-year PFS rate and one-year OS rate were 19.4 months, 69.8% and 90.2%, respectively. The toxicity profile was endurable. The most frequently reported drug-related grade III-IV side effects included hematologic adverse events, namely thrombocytopenia (14.3%) and neutropenia (46.4%). The study concluded that endostatin combined with GC chemotherapy exhibited feasibility, effective clinical response, survival advantages and tolerability in patients with metastatic NPC. Furthermore, it was recommended that endostatin combined with GC chemotherapy could emerge as a highly plausible therapy in patients with metastatic NPC.

In 2015, Guan et al. (retrospective study) tested the efficacy and toxicity of endostatin combined with CRT in 22 patients with locally recurrent NPC [21]. All patients (100%) achieved an objective clinical response as follows: 20 and two patients with complete and partial responses, respectively. The interval of follow-up ranged from four to 41 months (median 13 months). Four deaths occurred due to the following reasons: radiation-induced temporal lobe necrosis (n=1), NPC bleeding (n=1) and tumor metastasis (n=2). The one-year PFS, OS, loco-regional failure-free survival (LRFFS), distant metastasis-free survival (DMFS) rates were 92.3%, 93.3%, 89.3% and 90.0%, respectively. On the other hand, the two-year PFS, OS, LRFFS and DMFS rates were 52.7%, 66.4%, 78.1% and 78.8%, respectively. Serious acute grade III or more drug-related side effects comprised myelosuppression (n=3), mucositis (n=3) and cardiotoxicity (n=1). Conversely, serious delayed grade III or more drug-related side effects comprised nasopharyngeal mucosal necrosis (n=7) and radiation-induced encephalopathy (n=4). The study concluded that endostatin combined with CRT may be an effective regimen in patients with locally recurrent NPC; however, the conclusion of the study was severely limited by the small sample size of the research subjects.

In 2018, Hui et al. (phase II trial) gauged the efficacy and safety of axitinib in 40 recurrent or metastatic NPC patients who developed disease progression after a minimum of one line of previous platinum-based chemotherapy [22]. Only 37 patients were evaluable for response according to the response evaluation criteria in solid tumors (RECIST). Clinical benefit response was observed in 78.4% and 30.4% of patients at three months and six months, respectively. At three months, seven and 22 patients achieved partial responses and stable disease, respectively. The median follow-up time was 28.3 months. The median TTP, PFS and OS were five months, five months and 10.4 months, respectively. The one-year OS rate was 46.3%. In ascending order, the most frequently reported drug-related side effects (≥grade I) included diarrhea (33%), hypertension (38%), fatigue (40%) and equally hand-foot syndrome/hypothyroidism (50%). Specifically, hemorrhage-related adverse events occurred in seven (18%) patients as follows: six (15%) patients with grade I and one (3%) patient with grade II. In ascending order, the most frequently reported grade III adverse events comprised of equally pain/weight loss/diarrhea (5%) and hypertension (8%). No grade IV or higher adverse events occurred. The study concluded that single-agent axitinib was associated with the advantageous therapeutic index and fairly tolerable drug profile in heavily chemotherapy-pretreated patients with recurrent or metastatic NPC.

In 2018, Jin et al. (phase II trial) provided an update on a prior phase II trial that endeavored to evaluate the efficacy and safety of endostatin combined with GC chemotherapy [20,23]. The update included an analysis of 72 patients with metastatic NPC (28 old patients plus 44 new patients). The ORR was achieved in 56 (77.8%) patients, as follows: 40 (55.6%) and 16 (22.2%) patients with partial and complete responses, respectively. On the other hand, stable and progressive disease occurred in six (8.3%) and 10 (13.9%) patients, respectively. The interval of follow-up ranged from eight to 84 months (median 19.5 months). The median PFS and OS were 12 and 19.5 months, respectively. The one- and three-year PFS rates were 45.4% and 23.3%, respectively. Conversely, the one- and three-year OS rates were 87.4% and 31.9%, respectively. Overall, the chemotherapy regimen was well-endured. The most frequent grade III-IV hematologic side effects comprised neutropenia (59.8%) and leukopenia (54.1%). On the other hand, the most frequent grade III-IV non-hematologic side effects comprised liver dysfunction (11.1%), nausea/vomiting (4.2%) and anorexia (2.8%). No drug-related mortality was reported. The study concluded that the use of endostatin combined with GC chemotherapy was associated with beneficial clinical outcomes and acceptable toxicity profile in patients with metastatic NPC.

Table 1 shows a summary of the published literature on the role of VEGFR inhibitors in the management of patients with recurrent or metastatic NPC.

**Table 1:** Summary of the published literature on the role of vascular endothelial growth factor receptor (VEGFR) inhibitors in the management of patients with recurrent or metastatic nasopharyngeal carcinoma (NPC) * The objective response rate at six months. 5-FU: 5-fluorouracil; CRT: chemoradiotherapy; GC: gemcitabine and cisplatin; mon: month; NR: not reported; ORR: objective response rate including partial and complete responses; OS: overall survival; PFS: progression-free survival; Ref: reference; Retro: retrospective cohort study; TTP: time to progression

Ref	Authors	Year	Phase	Country	Patient number	Regimen	ORR (%)	Median TTP (mon)	Median PFS (mon)	Median OS (mon)	One-year PFS (%)	One-year OS (%)
[14]	Elser et al.	2007	II	Canada	7	Sorafenib	3.7	3.2	NR	7.7	NR	NR
[15]	Lim et al.	2011	II	Singapore	33	Pazopanib	6.1	4.4	NR	10.8	13	44.4
[16]	Hui et al.	2011	II	China	14	Sunitinib	28.6	4.4	3.5	10.5	NR	35.7
[18]	Huang et al.	2013	II	China	58	Famitinib	8.6	NR	3.2	NR	NR	NR
[19]	Xue et al.	2013	II	China	54	Sorafenib + cisplatin + 5-FU	77.8	NR	7.2	11.8	NR	NR
[20]	Jin et al.	2013	II	China	30	Endostatin + GC	85.7	NR	19.4	90.2	69.8	90.2
[21]	Guan et al.	2015	Retro	China	22	Endostatin + CRT	100	NR	NR	NR	92.3	93.3
[22]	Hui et al.	2018	II	China	40	Axitinib	30.4*	5	5	10.4	NR	46.3
[23]	Jin et al.	2018	II	China	72	Endostatin + GC	77.8	NR	12	19.5	45.4	87.4

Inhibition of epidermal growth factor receptor

Cetuximab, gefitinib and erlotinib are EGFR inhibitors. The EGF-EGFR interaction activates the Ras-Raf-MEK-ERK signaling pathway, which plays various important biological roles, such as: apoptosis, cell growth, cellular differentiation and cellular transformation [24-25]. Non-keratinizing NPC is characterized by high expression of EGFR [26], as well as EGFR gene amplification in pre-clinical NPC models and patients’ tumor samples [27]. EGFR expression in NPC is associated with poor clinical and survival outcomes [26]. Thus, molecular targeting of EGFR is a plausible therapeutic aim in recurrent and metastatic NPC.

In 2005, Chan et al. (phase II trial) examined the efficacy and toxicity of cetuximab and carboplatin (platinum-based cytotoxic agent) in 60 patients with recurrent or metastatic NPC who previously failed platinum-based chemotherapy [28]. Only 59 patients were evaluable for clinical response. Partial response, stable disease and progressive disease occurred in seven (11.7%), 29 (48.3%) and 23 (38.3%) patients, respectively. Thus, the ORR was 11.7%. The median TTP and OS were 2.9 months and 8.3 months, respectively. Almost all patients (n=58, 97%) experienced some sort of cetuximab-related side effects (≥grade I), and five (8%) patients experienced cetuximab-related side effects that resulted in cessation of cetuximab administration. Grade III-IV adverse events happened in 31 (51.7%) patients, and 19 (31.7%) of these patients were regarded to have cetuximab-related adverse events. No patient developed cetuximab-related hypersensitivity reaction. Two deaths were reported, and none of them was attributable to cetuximab therapy. The study concluded that cetuximab plus carboplatin appeared to exhibit clinical efficacy with tolerable toxicity drug profile in heavily chemotherapy-pretreated (platinum-based regimen) patients with recurrent or metastatic NPC.

In 2008, Chua et al. (phase II trial) explored the efficacy and toxicity of gefitinib in 19 patients with recurrent or metastatic NPC who were previously pretreated with at least two lines of chemotherapy regimens (including one line of platinum-based chemotherapy) [29]. The ORR was zero; no patient achieved a clinical partial or complete response. The median TTP and OS were four months and 16 months, respectively. Overall, the treatment was well-endured and only mild grade I-II drug-related adverse events were documented, as follows: equally anorexia and diarrhea (31.6%), fatigue (36.9%) and rash (68.5%). No grade III-IV drug-related adverse events or deaths were reported. The study concluded that gefitinib had endurable safety profile; however, it was associated with disadvantageously poor ORR in heavily chemotherapy-pretreated patients with recurrent or metastatic NPC. Moreover, it was suggested that gefitinib administration should be limited only to clinical trials.

In 2008, Ma et al. (phase II trial) investigated the efficacy and safety of gefitinib in 16 patients with recurrent or metastatic NPC who previously failed a platinum-based chemotherapy [30]. Only 15 patients were evaluable for clinical response. The ORR was zero; no patient achieved a clinical partial or complete response. Around 90% of patients experienced disease progression after receiving only two cycles of gefitinib. Only three (20%) patients achieved stable disease ranging from 2.8 to 8.5 months. The median TTP and OS were 2.7 months and 12 months, respectively. Collectively, the treatment was well-tolerated and only mild grade I-II drug-related toxicities were encountered, as follows (in ascending order): rash (60%), diarrhea (73%) and dry skin (93%). Grade III rash occurred in five (33%) patients and no interstitial lung toxicity or drug-related mortality were reported. The study concluded that despite gefitinib had favorable toxicity, it was associated with extremely weak anti-cancer activity in chemotherapy-pretreated patients with recurrent or metastatic NPC.

In 2012, You et al. (phase II trial) evaluated the efficacy and tolerability of erlotinib in 20 patients with recurrent or metastatic NPC. Erlotinib was employed as maintenance therapy after administration of gemcitabine-platinum chemotherapy regimen [31]. The study’s primary endpoint was TTP in patients without progressive disease after six cycles of gemcitabine-platinum chemotherapy and maintenance therapy with erlotinib. Only 13 patients met the primary endpoint of the study and the median TTP was 6.9 months. Overall, a sum of 15 patients received the erlotinib maintenance therapy, and only 12 patients were evaluable for erlotinib response. Among the 15 patients who received erlotinib maintenance therapy, 12 (75%) and 3 (25%) patients had progressive and stable disease, respectively. The mean interval for stable disease ranged from three to seven months (median 4.6 months). Anticipated side effects pertinent to gemcitabine-platinum regimen were detected. Concerning erlotinib safety profile, the drug was well-endured. No grade IV drug-related adverse events were reported. Anticipated erlotinib-related toxicities included grade I-III increased hepatic enzymes (42%) and grade I-II skin side effects (16%). The study concluded that erlotinib maintenance therapy did not yield favorable clinical benefits in gemcitabine-platinum-cotreated patients with recurrent or metastatic NPC.

In 2016, Xu et al. (retrospective study) assessed the efficacy and toxicity of cetuximab plus CRT in 30 patients with recurrent or metastatic NPC [32]. Chemotherapy regimens were diverse and included: (i) docetaxel plus cisplatin, (ii) docetaxel plus cisplatin plus 5-FU, (iii) gemcitabine plus cisplatin, and (iv) paclitaxel plus carboplatin. RT was administrated in an intensity-modulated method with a median dose of 60 Gy. A total of 21 (70%) patients achieved ORR, as follows: 18 patients with partial response and three patients with complete response. On the other hand, stable disease and progressive disease occurred in seven and two patients, respectively. The median interval of follow-up was 23.6 months and ranged from 3.3 to 53.4 months. The median TTP and OS were 12.2 months and 23.6 months, respectively. The two-year OS rate was 53.3%. The most commonly reported cetuximab-related side effects included equally rash/asthenia (93.2%), dry skin (76.7%) and oral mucositis (46.7%), all of which were of grade I-II. Overall, 19 deaths occurred at the time of study analysis, as follows: unknown reason (n=1), management-related adverse events (n=5) and cancer progression (n=13). The study concluded that cetuximab plus concurrent CRT was clinically effective with an acceptable toxicity profile in patients with recurrent or metastatic NPC.

In 2018, Ng et al. (phase II trial) scrutinized the efficacy and safety of induction chemotherapy (docetaxel, cisplatin, and 5-FU [TPF]) followed by maintenance chemotherapy (docetaxel and cetuximab) and IMRT in 33 patients with locally advanced recurrent NPC [33]. However, five patients discontinued the study due to an occurrence of ≥grade III adverse events and one patient died following the first cycle of TPF induction chemotherapy. Thus, 27 patients completed the management plan of the study, however, one patient died prior to study analysis. Thus, 26 patients were evaluable for response, and the clinical complete response and 3-year loco-regional control rates were 30.8% and 49.2%, respectively. The median duration of follow-up was 28.5 months. For all patients, the 3-year PFS was 35.7% whereas the 3-year OS rate was 63.8%. Collectively, five mortality-related events occurred, as follows: management-related epistaxis (n=2), management-related temporal lobe necrosis (n=1), acute induction chemotherapy-related following the first cycle (n=1) and acute biochemoradiotherapy-related (n=1). Grade III or more adverse events (in ascending order) comprised dysphagia (11.5%), soft tissue necrosis (15.4%), trismus (19.2%) and hearing loss (30.8%). Interestingly, temporal lobe necrosis occurred in eight patients. The study concluded that the presented management scheme (induction chemotherapy followed by docetaxel plus cetuximab and IMRT) could yield beneficial clinical outcomes. Nevertheless, the extremely unacceptable drug profile of induction chemotherapy and the increased incidence of temporal lobe necrosis should circumvent the administration of this presented management scheme in NPC patients outside clinical trials.

Table 2 shows a summary of the published literature on the role of EGFR inhibitors in the management of patients with recurrent or metastatic NPC.

**Table 2:** Summary of the published literature on the role of epidermal growth factor receptor (EGFR) inhibitors in the management of patients with recurrent or metastatic nasopharyngeal carcinoma (NPC) CRT: chemoradiotherapy; GP: Gemcitabine and paclitaxel; IMRT: intensity-modulated radiotherapy; mon: month; NR: not reported; ORR: objective response rate including partial and complete responses; OS: overall survival; PFS: progression-free survival; Ref: reference; Retro: retrospective cohort study; TPF: docetaxel, cisplatin, and 5-fluorouracil; TTP: time to progression

Ref	Authors	Year	Phase	Country	Patient number	Regimen	ORR (%)	Median TTP (mon)	Median PFS (mon)	Median OS (mon)	One-year PFS (%)	One-year OS (%)
[28]	Chan et al.	2005	II	China	60	Cetuximab + carboplatin	11.7	2.9	NR	8.3	NR	NR
[29]	Chua et al.	2008	II	China	19	Gefiinib	0	4	NR	16	NR	NR
[30]	Ma et al.	2008	II	China	16	Gefitinib	0	2.7	NR	12	NR	NR
[31]	You et al.	2012	II	Canada	20	Erlotinib after six cycles of GP	0	6.9	NR	Not reached	NR	NR
[32]	Xu et al.	2016	Retro	China	30	Cetuximab + CRT	70	12.2	NR	23.6	NR	NR
[33]	Ng et al.	2018	II	China	33	Neoadjuvant TPF + maintenance docetaxel and cetuximab + IMRT	30.8	NR	NR	NR	NR	NR

Discussion and interpretation

Table 1 and Table 2 display a summary of the published studies on molecular targeted therapy in patients with recurrent and metastatic NPC (n=15). There were slightly more studies on VEGFR inhibitors (n=9) than the EGFR inhibitors (n=6). The vast majority of studies originated from China (n=12), were phase II trials (n=13) and had small study sample sizes of less than 50 patients (n=11). Some studies employed molecular targeted therapy as stand-alone drugs (second-line agents or beyond, n=7), whereas a few studies combined molecular targeted therapy with cytotoxic agents and/or RT and/or concurrent CRT (n=8). Roughly, the bulk of studies reported median TTP ranging from 2.7 to 12.2 months, PFS ranging from 3.2 to 19.4 months and OS ranging from 7.7 to 23.6 months.

Overall, molecular targeted therapy against VEGF/VEGFR and EGF/EGFR does not seem to offer valuable clinical and survival benefits to patients with recurrent and metastatic NPC. Moreover, the small study sample sizes, lack of phase III trials and short duration of follow-up are some of the major shortcomings of the studies reporting molecular targeted therapy in patients with recurrent and metastatic NPC. Furthermore, the absence of evaluation of quality-of-life before and after administration of molecular targeted therapy is an additional shortcoming. All these shortcomings, collectively, contribute to a limitation in withdrawing concrete conclusions. Thus, as it stands now, the role of molecular targeted therapy in patients with recurrent and metastatic NPC remains to be further investigated.

There are a number of ongoing studies that are currently investigating angiogenesis inhibition targets (mainly VEGF and endostatin). For example, NCT02250599 is a Chinese phase III study in which patients with untreated metastatic NPC will be randomized to receive cytotoxic chemotherapy carboplatin and paclitaxel (TC) alone or TC plus bevacizumab. NCT02636231 is a Chinese phase II study in which patients with untreated locally recurrent NPC will be randomized to receive IMRT alone or IMRT plus endostar (recombinant endostatin). NCT02590133 is a Chinese phase II study in which patients with refractory NPC will be randomized to receive nedaplatin plus low-dose 5-FU infusion alone (group one) or nedaplatin plus low-dose 5-FU infusion plus endostar (group two).

Nowadays, an array of new genes and growth factor signaling pathways are currently under investigation as potential targets for novel targeted therapy. Such examples include the phosphoinositide 3-kinase (PI3K), nuclear factor kappa B (NF-κB) and cyclin-dependent kinase-4 (CDK4) pathways [34]. These pathways have been shown to contribute key roles in the pathogenesis of NPC, and thus, targeting such pathways and may emerge as plausible molecular therapeutic targets.

At the present time, the first-line therapy for recurrent and metastatic NPC is a platinum-based doublet chemotherapy regimen, particularly cisplatin plus gemcitabine [2, 35]. For patients with recurrent and metastatic NPC who previously failed platinum-based doublet chemotherapy, a re-challenge (second-line therapy) with a platinum-based doublet chemotherapy regimen remains a plausible thought [2]. As it stands now, the role of molecular targeted therapy is not convincing and should be re-evaluated [2, 6]. It may be interesting to consider the combination of cytotoxic chemotherapy and molecular targeted therapy in patients with recurrent or metastatic NPC [2, 6]. Recently, the use of immunotherapy in the management of recurrent or metastatic NPC has evolved gradually and gained popularity [2, 6]. The modalities of immunotherapy include adoptive transfer of cytotoxic T cells (CTLs) specific for EBV antigens [36], therapeutic EBV vaccination [37-38] and immune checkpoint inhibitors [39-40]. However, despite the initial findings are promising with comparatively safe toxicity profile, the vast majority of these approaches harbor limitations, such as: being experimental in design, phase II trials, bear high therapy-related costs and confined only to super-specialized tertiary healthcare centers that are well-funded and possess comprehensive laboratory infrastructures [2, 6]. Currently, there are two ongoing randomized trials investigating pembrolizumab (NCT02611960) or PDR001 (NCT02605967) versus the gold standard chemotherapy in patients with recurrent or metastatic NPC that progressed after pre-treatment with platinum-based chemotherapy.

## Conclusions

In patients with recurrent and metastatic NPC, molecular targeted therapy does not seem to offer clinical and survival benefits. Future research directions may include a combination of cytotoxic chemotherapy ± old/new molecular targeted therapy ± immunotherapy. An optimal approach in the management of recurrent and metastatic NPC should be directed toward: (i) induction of favorable tumor response (clinically, radiologically and histologically), (ii) minimal drug-related adverse events, and (iii) reduction in the rate of further loco-regional or distant recurrences.
